# Malposition of peripherally inserted central catheter: Experience from 3,012 patients with cancer

**DOI:** 10.3892/etm.2013.1267

**Published:** 2013-08-20

**Authors:** LINPING SONG, HUI LI

**Affiliations:** Department of Oncology, The First Affiliated Hospital of Chinese PLA General Hospital, Beijing 100048, P.R. China

**Keywords:** peripherally inserted central catheter, misplaced catheter, cancer patients

## Abstract

The aim of this study was to observe and analyze the causes of misplacement of peripherally inserted central catheters (PICCs) in patients with cancer. A total of 3,012 patients who underwent insertion of a PICC were reviewed from August 2000 to March 2012. The locations of the tube tips were recorded by chest X-ray examination. Malposition of the PICC was observed in 237 cases (7.87%), with the most frequently occurring site of misplacement being the jugular vein, followed by the axillary vein. By taking different remedies, all the malpositioned PICCs were relocated back to the superior vena cava or subclavian vein. In order to ensure the safe usage of PICCs, strict placement guidelines, skilled and experienced healthcare professionals and the cooperation of the patient is necessary.

## Introduction

Peripherally inserted central catheter (PICC) technology has been widely used to provide long-term intravascular access in children and adults. With such frequent application in clinical practice, an increasing number of catheter complications have been observed. The incidence of malpositioned catheter is 5–31%, including malpositioned catheter, catheter slide or extrusion, and catheter drift (1). Malpositioned catheters result in discomfort and pain to the patients, and also induce serious consequences, such as loss of usage (2).

It has been shown that a central tip location for the PICC is essential to minimize the risk of complications. However, different catheter tip locations may generate different symptoms in patients (3,4). If the tip is inserted into the heart, precordial discomfort, arrhythmia, cardiac tamponade and heart valve damage may occur in the patient; if the catheter remains in a peripheral vein, it may induce swelling, pain, fluid in the limb of the patient and discomfort and pain at the site where the catheter tip attaches to the vessel wall. In addition, the placement of the catheter tip in the jugular vein may result in discomfort, difficulty in turning the head and neck soreness occurring in the affected side (5). Therefore, the establishment of a protocol to avoid the misplacement of the PICC is very important in clinical practice. In the present study, we monitored a total of 3,012 patients with cancer who underwent insertion of a PICC and summarized the data from these patients.

## Materials and methods

### Patients

From August 2000 to March 2012, a total of 3,012 cases underwent insertion of a PICC (1,590 adult males, 1,121 adult females and 301 children; age range, 1∼94 years; median age: 52 years). All the patients received chemotherapy or nutritional support.

### PICC placement

Three types of 4F catheters were prepared for PICC insertion, with the selection dependent on the patient: i) 3-position Groshong valve type catheter, 60 cm in length (C.R. Bard, Inc., Murray Hill, NJ, USA); ii) catheter with a length of 55 cm (Arrow International, Inc., Reading, PA, USA); iii) catheter with a length of 45 cm (B. Braun Melsungen AG, Melsungen, Germany). The catheters were inserted through the basilic vein in 1,805 cases, through the median cubital vein in 791 cases and through the cephalic vein in 416 cases.

All the catheters were placed by PICC nurses who had undergone specialized training. Prior to the PICC insertion, the catheter length was estimated by the professional carrying out the insertion (6). The insertion of the PICC was performed using aseptic techniques and procedures by the bedside. A chest X-ray was obtained following the procedure. PICC tips were defined as central if they resided anywhere within the superior vena cava (SVC). All X-ray images were read by a radiologist to determine the PICC tip locations.

## Results

**Figure 1.**
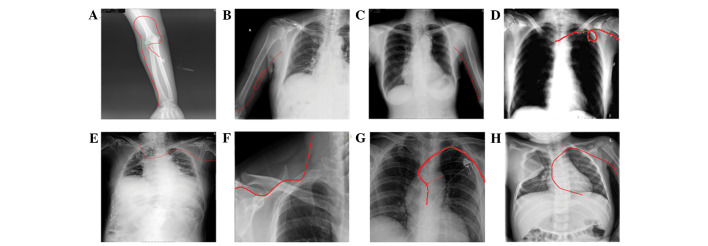
Typical malpositions of peripherally inserted central catheters (PICCs): (A) Bending in the forearm; (B) bending in the basilic vein; (C) stopping at the armpit; (D) bending in the subclavian vein; (E) entering the contralateral subclavian vein; (F) entering the jugular vein; (G) entering the azygos vein; (H) entering the right ventricle. Red lines indicate the location of the PICCs.

The rate of successful initial placement was 94.6% (2848/3012). Catheter malposition occurred in 237 out of the 3,012 patients (7.87%). X-ray examination clearly showed the malposition of PICC in forearm, basilic vein and armpit, which is indicated by red lines in Fig. 1. The most frequently occurring site of misplacement was the jugular vein which occurred in 99 patients (99/327, 30.3%), followed by the axillary vein (65/327, 19.9%) and brachial vein (25/327, 7.6%). Catheter prolapse was another noteworthy complication following PICC insertion (Table I).

## Discussion

PICCs have been widely used in clinical practice for several decades and have been shown to be a safe and convenient means of administering chemical drugs and parenteral hyperalimentation (7,8). When the catheter does not reach the appropriate location within the vena cava, it is considered to be misplaced. This complication is very common in clinical practice. If the catheter is difficult to thread or to insert to the premeasured depth, blood withdrawal is difficult, the catheter flushes with resistance or removal of the stylet is difficult, this is an indication that malpositioning may have occurred (9). An X-ray examination following PICC insertion is necessary to identify whether the catheter is misplaced.

It has been reported that the internal jugular vein is the most common malpositioning site following catheter insertion into the basilic vein, while the axillary and basilic veins are the most common malpositioning sites following catheter insertion into the cephalic vein. The insertion of catheters through the saphenous or other leg veins may lead to entry into the ascending lumbar vein (10,11), while catheter insertion into the scalp may result in entry into the intracranial veins or tissue and thoracic veins (12).

The clinical skill and experience of the healthcare professional is important to ensure successful catheter placement. Furthermore, a knowledge of venous anatomy, which may aid in the selection of a suitable vein and a suitable catheter for insertion, is essential. It is also important for the catheter to be inserted slowly, to allow the blood returning to the heart to carry the catheter to the vena cava. Rapid threading of the catheter may increase the risk of malposition. In addition, the position of the patient may impact the PICC insertion, with misplacement potentially occurring if it is not possible to position the patient’s jaw close to the shoulder.

Catheter care and maintenance is an important issue requiring the efforts of nurses and patients. There is a requirement for all nurses caring for patients with a PICC to be knowledgeable about the effective management of the catheter, in order to prolong the indwelling time of the catheter and to prevent complications and injury to the patients. Using a team of caregivers with trouble-shooting expertise and the ability to change dressings and repair catheters has been observed to enhance success with PICCs (13). To ensure that the catheter is kept in good condition, it is of great importance to educate the patients following the insertion of the PICC. Even after a successful placement, inappropriate movements of the patients and high intracranial pressure in patients with severe nausea, vomiting, hiccups and constipation may result in the malpositioning of the catheter tip. Certain additional factors may also result in the malpositioning of the catheter, such as patient stress and a cooler surrounding environment.

In conclusion, the most frequently occurring site of catheter misplacement is the jugular vein, followed by the axillary vein. Many factors may lead to the malposition of PICCs. The appropriate placement of PICCs requires the combined efforts of healthcare professionals and patients.

## Figures and Tables

**Table I.** Two hundred and thirty-seven cases of malpositioned PICC.

Location	During PICC insertion	Following PICC insertion
Right atrium	2	1
Right ventricle	1	0
Radial vein	1	0
Jugular vein	77	22
Brachial vein	19	6
Fold in basilic vein	3	0
Fold from basilic vein back into cephalic vein	2	0
Fold from cephalic vein back into basilic vein	1	0
Fold in cephalic vein	1	0
Catheter prolapse	0	19
Axillary vein	45	20
Contralateral subclavian vein	7	5
Azygos vein	5	0
Total	164	73

PICC, peripherally inserted central catheter.

## References

[1] Ryder MA (1993). Peripherally inserted central venous catheters. Nurs Clin North Am.

[2] Walshe LJ, Malak SF, Eagan J, Sepkowitz KA (2002). Complication rates among cancer patients with peripherally inserted central catheters. J Clin Oncol.

[3] Yamamoto AJ, Solomon JA, Soulen MC (2002). Sutureless securement device reduces complications of peripherally inserted central venous catheters. J Vasc Interv Radiol.

[4] Kearns PJ, Coleman S, Wehner JH (1996). Complications of long arm-catheters: a randomized trial of central vs peripheral tip location. JPEN J Parenter Enteral Nutr.

[5] James L, Bledsoe L, Hadaway LC (1993). A retrospective look at tip location and complications of peripherally inserted central catheter lines. J Intraven Nurs.

[6] Marcy PY (2008). Central venous access: techniques and indications in oncology. Eur Radiol.

[7] Tian G, Zhu Y, Qi L, Guo F, Xu H (2010). Efficacy of multifaceted interventions in reducing complications of peripherally inserted central catheter in adult oncology patients. Support Care Cancer.

[8] Cunningham RS, Ravikumar TS (1995). A review of peripherally inserted central venous catheters in oncology patients. Surg Oncol Clin N Am.

[9] Aladangady N, Roy R, Costeloe KL (2005). The cobweb sign: percutaneous silastic long line tip placement in tributaries of superficial veins. J Perinatol.

[10] Clarke P, Wadhawan R, Smyth J, Emmerson AJ (2003). Parenteral nutrition solution retrieved by lumbar puncture following left saphenous vein catheterization. J Paediatr Child Health.

[11] De A, Imam A (2005). Long line complication: accidental cannulation of ascending lumbar vein. Arch Dis Child Fetal Neonatal Ed.

[12] Andersen C, Hart J, Vemgal P, Harrison C (2005). Prospective evaluation of a multi-factorial prevention strategy on the impact of nosocomial infection in very-low-birthweight infants. J Hosp Infect.

[13] Golombek SG, Rohan AJ, Parvez B, Salice AL, LaGamma EF (2002). “Proactive” management of percutaneously inserted central catheters results in decreased incidence of infection in the ELBW population. J Perinatol.

